# SpiB regulates the expression of B-cell-related genes and increases the longevity of memory B cells

**DOI:** 10.3389/fimmu.2023.1250719

**Published:** 2023-10-27

**Authors:** Shu Horiuchi, Takuya Koike, Hirofumi Takebuchi, Katsuaki Hoshino, Izumi Sasaki, Yuri Fukuda-Ohta, Tsuneyasu Kaisho, Daisuke Kitamura

**Affiliations:** ^1^ Division of Cancer Cell Biology, Research Institute for Biomedical Sciences, Tokyo University of Science, Noda, Chiba, Japan; ^2^ Department of Immunology, Faculty of Medicine, Kagawa University, Miki-cho, Kagawa, Japan; ^3^ Laboratory for Human Disease Models, RIKEN Center for Integrative Medical Sciences, Yokohama, Kanagawa, Japan; ^4^ Department of Immunology, Institute of Advanced Medicine, Wakayama Medical University, Wakayama, Japan

**Keywords:** memory B cell, germinal center B cell, SpiB, apoptosis, autophagy, humoral immune response, SpiB conditional knockout mice

## Abstract

Generation of memory B cells is one of the key features of adaptive immunity as they respond rapidly to re-exposure to the antigen and generate functional antibodies. Although the functions of memory B cells are becoming clearer, the regulation of memory B cell generation and maintenance is still not well understood. Here we found that transcription factor SpiB is expressed in some germinal center (GC) B cells and memory B cells and participates in the maintenance of memory B cells. Overexpression and knockdown analyses revealed that SpiB suppresses plasma cell differentiation by suppressing the expression of Blimp1 while inducing Bach2 in the *in-vitro*-induced germinal center B (iGB) cell culture system, and that SpiB facilitates *in-vivo* appearance of memory-like B cells derived from the iGB cells. Further analysis in IgG1^+^ cell-specific SpiB conditional knockout (cKO) mice showed that function of SpiB is critical for the generation of late memory B cells but not early memory B cells or GC B cells. Gene expression analysis suggested that SpiB-dependent suppression of plasma cell differentiation is independent of the expression of Bach2. We further revealed that SpiB upregulates anti-apoptosis and autophagy genes to control the survival of memory B cells. These findings indicate the function of SpiB in the generation of long-lasting memory B cells to maintain humoral memory.

## Highlights

1. GC and memory B cells express SpiB and SpiB expression is induced by CD40 stimulation *in vitro*.
2. SpiB suppresses plasma cell differentiation while inducing the expression of Bach2 *in vitro*.
3. SpiB overexpression enhances and SpiB knockdown diminishes B_mem_-like cell generation.
4. SpiB Cγ1-cKO mice generates less IgG1^+^ late B_mem_ cells with diminished memory antibody response.
5. SpiB suppresses plasma cell differentiation independent of Bach2.
6. SpiB Cγ1-cKO B cells express lower levels of anti-apoptosis and autophagy genes with less ability to survive.

## 1 Introduction

Upon immunization with T-cell-dependent (TD) antigens, antigen-activated B cells undergo proliferation and class switching of their B cell receptor (BCR) and differentiate into effector B cell populations. Some B cells differentiate to short-lived plasma cells (PCs) that produce low affinity antibodies at the early stage of the immune response. Other B cells differentiate to germinal center (GC) B cells to form GCs. In GCs, B cells undergo somatic hypermutation (SHM) in variable regions of immunoglobulin genes, encoding BCR and antibodies, and selection based on the affinity of the resultant BCR to the antigen. Among the selected B cells, those expressing BCR with relatively higher affinity differentiate into long-lived PCs (LLPCs) whereas those with relatively lower affinity to memory B (B_mem_) cells. In addition, B_mem_ cells are also generated independently of GCs. LLPCs and B_mem_ cells, of either GC-experienced or not, contribute to long-term humoral immunity (1–3).

B_mem_ cells are maintained in the body for a long time and activated rapidly when re-exposed to the primary antigen, partly owing to IL-9 autocrine stimulation (4, 5). The typical recall response for class-switched B_mem_ cells is to produce a large amount of antigen-specific antibodies in a short time. A small proportion of the class-switched B_mem_ cells and most IgM^+^ B_mem_ cells differentiate to GC B cells to undergo further SHM and affinity maturation during the recall response. Although regulatory mechanisms of the B_mem_ cell recall response through BCR and other receptors are becoming clearer (4), the mechanisms for B_mem_ cell development, including external signals and transcription factors that induce differentiation to B_mem_ cells, are largely unknown. Bach2 is a transcriptional suppressor that regulates the expression of *Blimp1*, a gene encoding a transcription factor known to induce PC development. Bach2 was shown to be required for GC B-cell differentiation toward B_mem_ cells albeit in a specific genetic background, namely Blimp1-deficiency (6).

To elucidate the mechanisms for B_mem_ cell development, we established an experimental system in which naive B cells can be committed to either B_mem_ cells or PCs *in vitro*. In this system, naïve B cells are propagated on the feeder cells expressing exogenous CD40L and BAFF (termed 40LB) in the presence of IL-4 for the first 4-5 days. This culture allows massive expansion of B cells expressing GC B-cell markers, which we call ‘induced GC B (iGB)-4 cells’, and efficient class switching either to IgG1 or IgE. The following culture with IL-21 allows further proliferation of iGB cells (now called iGB-21) for another 4-5 days, gradually increasing the proportion of CD138^+^ plasmablasts. Importantly, when transferred into irradiated wild type mice, the iGB-4 cells differentiate into memory-like B cells termed “induced memory B (iMB) cells”, which are phenotypically and functionally equivalent to *bona fide* B_mem_ cells and survive more than 2 months in the spleens and lymph nodes of the recipient mice. Notably, IgG1^+^ and IgM^+^, but not IgE^+^, iGB-4 cells become iMB cells *in vivo*. In contrast, iGB-21 cells fail to develop into iMB cells but instead differentiate into PCs in the bone marrow of the recipients. The iGB cells tertiary cultured for another 2 days without any cytokines (iGB-m) partly regain the ability to form iMB cells *in vivo* (7). From the data set of microarray analysis comparing iMB cells and naive follicular B cells, we selected the genes expressed in excess in iMB cells, and further selected those expressed at a higher level in IgG1^+^ iGB-4 cells than in IgG1^+^ iGB-21 cells, and not in IgE^+^ iGB-4/iGB-21 cells, aiming to identify the genes that regulate B_mem_ cell development. Among the genes selected in this approach, we focused on *SpiB.*

SpiB is an ETS family transcription factor expressed in various types of immune cells (8–10). In B cells, it has been reported to regulate the differentiation of pre-B cells in the bone marrow and follicular B cells (11, 12). SpiB is also required for proper BCR signaling (13) and mature B cell survival, although being compensated by PU.1 (12), and for sustaining GC B cells in the primary, and antibody production in the secondary, TD immune response (14, 15). In an *in vitro* study, SpiB suppresses PC differentiation in human and mice (16, 17). However, the role of SpiB in B cells after the generation of the GC is unknown.

Here we show our finding that SpiB is critical for B_mem_ cell maintenance. *SpiB* mRNA is expressed in GC B cells and B_mem_ cells, and its expression is upregulated by CD40 stimulation. Overexpression and knockdown of SpiB in iGB cells revealed that SpiB induces the expression of *Bach2* and suppresses the induction of *Blimp1* expression and the differentiation of CD138^+^ PCs, independently of Bach2, and that SpiB facilitates iMB cell formation from iGB-4 cells *in vivo*. A conditional mouse strain that lacked SpiB only in IgG1^+^ B cells (SpiB cKO) exhibited defective formation of IgG1^+^ late B_mem_ cells after primary immunization and attenuated IgG1 recall responses upon the secondary challenge. Additionally, B_mem_ cells in the SpiB cKO mice showed lower expression of genes required for anti-apoptosis and autophagy, suggesting that SpiB is critical for long-term survival of B_mem_ cells.

## 2 Materials and methods

### 2.1 Mice and immunization

C57BL/6 NCrSlc (B6) mice were purchased from Sankyo Labo Service. All the following mouse strains were backcrossed to B6 or congenic B6 CD45.1^+^ mice: *SpiB* ^f/f^ (see below), *Blimp1* ^f/f^, Blimp1-GFP (18), and *Bach2* ^-/-^ (19). *SpiB* ^f/f^ and *Blimp1* ^f/f^ mice were crossed with Cγ1-cre (20) and CD19-cre mice (21), respectively, to generate each conditional knockout strain. As the control for the *SpiB* ^f/f^ Cγ1-cre (here called ‘SpiB cKO’) mice, *SpiB* ^+/+^ Cγ1-cre (here called ‘SpiB WT’) mice were used. Sex-matched 7-12 week-old mice were immunized i.p. with 100 μg of 4-hydroxy-3-nitrophenyl acetyl (NP)_32_-chicken gamma globulin (CGG) in alum or NP-conjugated sheep red blood cell (SRBC) where indicated. Mice were maintained in the Tokyo University of Science mouse facility under specific pathogen-free conditions. Mouse procedures were performed under protocols approved by the Animal Care and Use Committee of Tokyo University of Science (Approval No S13009).

### 2.2 Generation of Spib-floxed mice

To generate the *Spib*-floxed (*SpiB* ^f/f^) mice, a targeting vector was designed to delete exon 6 encoding the Ets domain of Spi-B. To construct the targeting vector, C57BL/6N mouse genomic DNA fragments were amplified by PCR using KOD -plus- DNA polymerase (Toyobo, Osaka, Japan). An 8.0 kbp long arm containing exon 6, which has an insertion of a loxP site in the intron downstream of the exon 6, was connected to an MC1 promoter-driven neomycin resistance gene (neo) cassette flanked by two FRT sites and a loxP site. Then, a 1.9 kbp short arm containing exons 3-5 was ligated upstream of the neo cassette. A herpes simplex virus thymidine kinase gene (HSV-TK) was attached at the downstream end of the long arm. The targeting vector was linearized with SalI and electroporated into the Bruce4 ES cells. Clones with resistance to G418 and gancyclovir were screened for homologous recombination by PCR and confirmed by Southern blot analysis. ES cell clones carrying the targeted allele were used to generate *Spib* mutant mice. To delete the neo cassette flanked by two FRT sites, the *Spib*-targeted mice were intercrossed with C57BL/6-Tg(CAG-flpe)36Ito/ItoRbrc (RBRC01834) (RIKEN BioResource Research Center, Japan) (**Supplementary Figure 1A**).

Genotyping of *Spib* mutant mice was performed by PCR using the primers listed below. PCR using primers #1 and #2 generates 301bp and 475 bp bands in the wild-type and floxed alleles, respectively. PCR using primers #1 and #3 generates a 454 bp band in the deleted allele. Although PCR using primers #1 and #3 should also generate a band with larger than 2700 bp in both wild-type and floxed alleles, it was too large to be detected (**Supplementary Figures 1B, C**).

Primer #1: 5’-AACCAGGCCCAACTCCATTGTGAAAAC-3’.

Primer #2: 5’-GCCAGCTTCTGATACGTCATGCGCTTG-3’.

Primer #3: 5’-AGTCCTGTCACACCATTGGTTGCAGTG-3’.

### 2.3 Flow cytometry

Single-cell suspensions from spleens were depleted of RBCs by ammonium chloride lysis, incubated with mAb to FcγRII/III (2.4G2; BD Pharmingen) to block FcγRs and were stained for 30 min on ice in PBS containing 0.5% BSA, 2 mM EDTA, 0.05% sodium azide with various combinations of antibodies and reagents. Intracellular SpiB staining was performed with FoxP3 Staining Buffer Set according to the manufacturer’s protocol (eBioscience) with anti-SpiB sheep polyclonal antibody (R&D systems, AF7204) followed by Alexa 647-labeled donkey anti-sheep IgG (Invitrogen, A-21448). All samples were analyzed by either FACS Callibur or FACS Canto II (BD Biosciences). The data were analyzed by FlowJo (Tree Star).

### 2.4 Cell culture and purification

Splenic naïve B cells were purified as described previously (7). B cells were cultured in RPMI-1640 medium (Wako) supplemented with 10% FCS, 10 mM HEPES, 1 mM sodium pyruvate, 5.5 × 10^−5^ M 2-ME, 100 U/ml penicillin, and 100 μg/ml streptomycin (GIBCO). For iGB cell culture, naive B cells were cultured on 40LB feeder cells with IL-4 (10 ng/ml) or IL-21 (10 ng/ml) where indicated. To purify iGB cells or to change the culture condition, 40LB feeder cells were removed from the total cells of iGB cultures by anti-H-2Kd (BioLegend) Ab, Streptavidin Particle Plus DM and the IMag system (BD Biosciences).

### 2.5 Cell sorting

Early B_mem_ and GC B cells were purified from spleen of the mice immunized with NP-SRBC, 7 or 14 days previously, as follows: splenocytes were stained with anti-CD19, anti-CD38, anti-GL7, and NP-BSA-biotin, followed by fluorescence-labeled streptavidin. B_mem_ cells (CD19^+^ NP^+^ CD38^+^ GL7^–^) and GC B cells (CD19^+^ NP^+^ CD38^–^ GL7^+^) were sorted by FACS AriaII. For iGB cells, cultured cells depleted of 40LB feeder cells as described above, were stained with anti-IgG and anti-IgE and sorted by FACS Aria II.

### 2.6 Retroviral transduction

Retroviruses were produced in Plat-E cells transfected with pMXs-based vectors with Fugene HD (Promega) or PEI “Max” (Mw 40,000; Polysciences). The virus-containing supernatant was collected 2 days after transfection and added to iGB cells cultured for 2 days previously. Cells were spin-infected at 2,000 rpm, room temperature for 90 minutes with 10 μg/ml DOTAP Liposomal Transfection Reagent (Roche) and IL-4 (10 ng/ml). One day later, cells were collected and cultured on new 40LB feeder layers with IL-4 for further culture.

### 2.7 Adoptive transfer for iMB cell generation

iMB cells were generated as previously described (7). Retrovirally transduced iGB cells from CD45.1^+^ B6 mice were collected by removing 40LB feeder cells with anti-H-2Kd Ab (BioLegend), Streptavidin Particle Plus DM, and the IMag system (BD Biosciences). The iGB cells were adoptively transferred to γ-irradiated (6.5Gy) B6 mice (CD45.2^+^). Splenocytes were harvested from the recipient mice after 2 weeks or later for further analysis.

### 2.8 Quantitative RT-PCR

The procedures for RNA extraction and reverse transcription to cDNA have been described previously (7). Quantitative real-time PCR was performed with a 7500 fast Real-time PCR system (Applied Biosystems). Gene expression levels were determined by the relative standard curve method and normalized to that of either *Gapdh* or *18S rRNA*.

### 2.9 ELISA

Total and high affinity NP-specific IgG1 was detected by the method described previously (7). NP_13.6_-BSA or NP_2.7_-BSA for total or high affinity antibodies, respectively, was coated on 96 well flat bottom plate. Bound Abs were detected by HRP-conjugated goat anti-mouse IgG1 Ab (Southern Biotechnology) and TMB substrate (Sigma).

## 3 Results

### 3.1 GC and memory B cells express SpiB and SpiB expression is induced by CD40 stimulation *in vitro*

**Figure 1:**
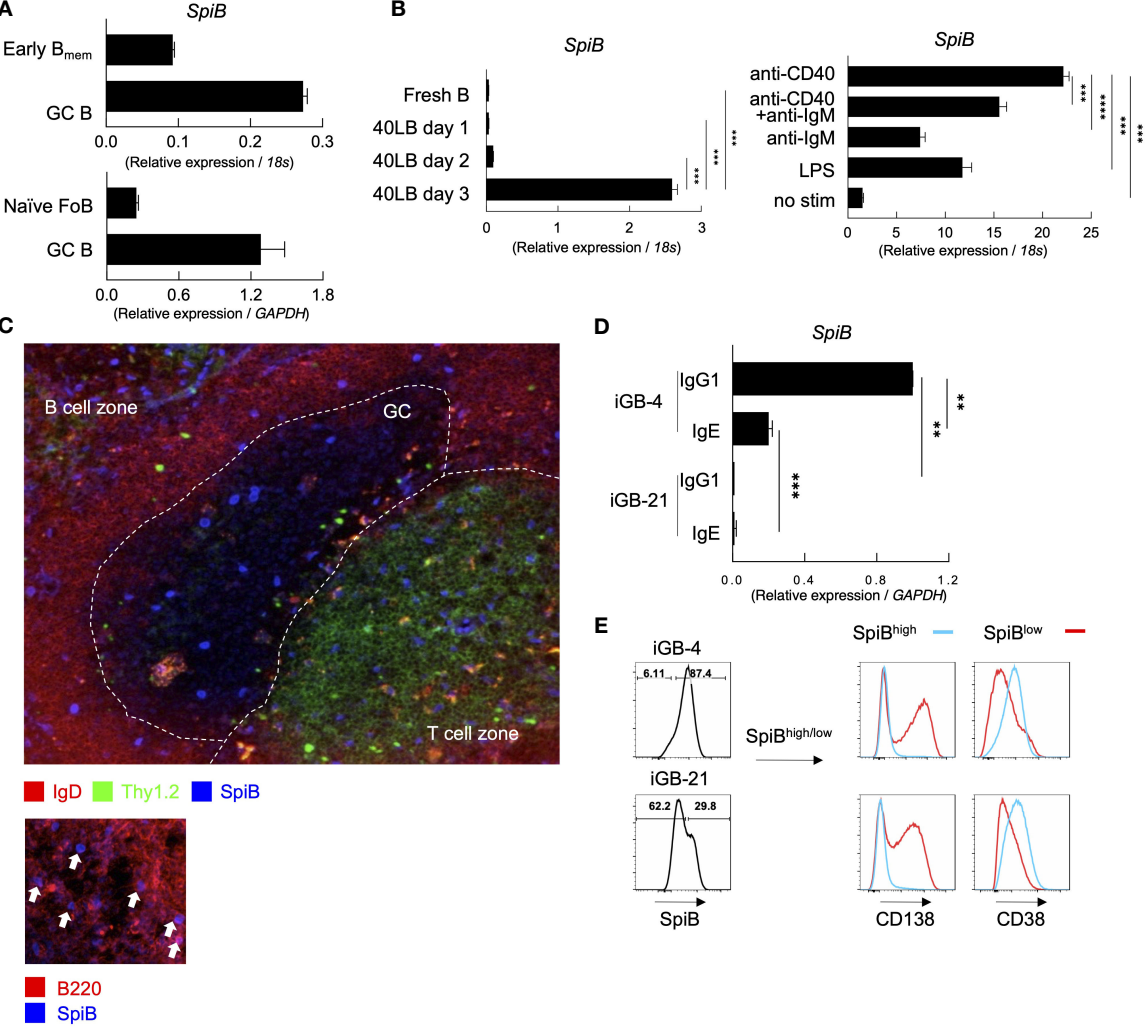
SpiB is expressed in GC B and B_mem_ cells and induced by CD40 stimulation *in vitro.* **(A)** *SpiB* mRNA expression in B cell subsets from mouse spleen collected 14 days post NP-CGG immunization. B cells were sorted as CD19^+^ Igκ^-^ and NP^+^ CD38^+^ GL7^-^ (early B_mem_ cells), NP^+^ CD38^-^GL7^+^ (GC B cells), or CD19^+^ CD21^lo^ CD23^hi^ AA4.1^-^ [naive follicular B (FoB) cells]. Comparison of early B_mem_ and GC B cells (top) and naïve FoB and GC B cells (bottom) are shown. **(B)** *SpiB* mRNA expression in B cells cultured on 40LB feeder cells for 0-3 days (left) and B cells stimulated with the indicated antibodies or LPS (right). **(C)** Immunofluorescence microscopy of frozen sections of spleens from mice immunized with NP-SRBC 7 days previously. The sections were stained for SpiB (blue), IgD (red), and Thy1.2 (green) (top), or for SpiB (blue) and B220 (red) (bottom). **(D)** *SpiB* mRNA expression in sorted IgG1^+^ and IgE^+^ from iGB cells cultured with IL-4 (iGB-4) for 4 days and IL-21 (iGB-21) for the following 4 days. **(E)** FCM analysis of the expression of CD138 and CD38 in SpiB^high^ (blue) and SpiB^low^ (red) iGB-4 and iGB-21 cells. SpiB staining profile and gating of iGB-4 and iGB-21 cells are shown on the left and the expression of CD138 and CD38 on the gated fractions are on the right. **p < 0.01, ***p < 0.001, ****p < 0.0001.

To understand the regulation of B_mem_ cells by transcription factors, we analyzed the gene-expression profiles of induced memory-like B (iMB) cells and compared the result with publicly available gene expression data (ImmGen). We noticed that mRNA for the transcription factor SpiB was expressed selectively high in B_mem_ cells among B cells, while hardly expressed in plasmablasts and PCs. In contrast, Bcl6 was expressed substantially lower in B_mem_ cells than in GC B cells (**Supplementary Figure 2A**). To confirm these expression patterns, we analyzed the mRNA levels of *SpiB*, *Bach2* and *Bcl6* in early B_mem_ and GC B cells from mice immunized with NP-CGG, and naïve follicular B cells from unimmunized mice. In slight contrast to the ImmGen data, *SpiB* expression in the early B_mem_ cells was lower than that in GC B cells, but appeared to be higher than that in follicular B cells. These expression patterns are similar to those of *Bach2* and *Bcl6* (**Figure 1A**; **Supplementary Figures 2B, C**).

Our previous finding proposed that the quantity of CD40 stimulation on B cells during the primary response is a key factor in regulating B_mem_ cells (22). Additionally, SpiB is reported to be induced by OBF-1 which is upregulated by CD40 signaling (23, 24). Thus, we assessed whether CD40 stimulation induces the expression of *SpiB* in B cells. By culturing naïve B cells on feeder cells expressing CD40L and BAFF (40LB) without any cytokines, the expression of *SpiB* was induced after 3 days (**Figure 1B**). We then tested other stimuli known to activate naïve B cells, namely, anti-CD40 or/and anti-IgM antibodies, or LPS, and observed that anti-CD40 induced the highest levels of *SpiB* expression among these stimuli, and no synergistic effect was seen between anti-CD40 and anti-IgM antibodies (**Figure 1B**). These results indicate that CD40 stimulation is sufficient to induce SpiB expression in B cells. Immunofluorescence microscopy analysis of spleens from mice immunized with NP-SRBC 7 days earlier showed that a small number of discernible SpiB^+^ cells were scattered in GC, B cell follicles, and T cell zones in the white pulp, while SpiB expression was undetectable in the vast majority of GC and follicular B cells (**Figure 1C**, top). At a closer look, nuclear SpiB^+^ cells in the B cell follicle were also B220^+^ (**Figure 1C**, bottom), either IgD^+^ or IgD^–^, and those in the GC and T cell zones were discernible as IgD^–^ (**Figure 1**, top). Together with our mRNA expression data and previous reports showing that *SpiB* mRNA expression is limited to B cells and plasmacytoid dendritic cells (9; ImmGen), these results suggest that a significant level of SpiB is expressed in selected GC B cells, class-switched or unswitched B_mem_ cells in the B cell follicle, and plasmacytoid dendritic cells in the T cell zone.

It has been shown that SpiB suppresses the expression of Blimp1 in human cells (17) and potentially in mice (16, 25). To further infer the role of SpiB in the late B-cell development, we analyzed the expression of SpiB in B cells cultured in the iGB system (7). The expression of *SpiB* mRNA was readily detected in IgG1^+^ B cells cultured on 40LB feeder cells with IL-4 (iGB-4 cells), and in IgE^+^ iGB-4 cells albeit at a reduced level, but declined to an almost undetectable level following culture with IL-21 (iGB-21 cells) (**Figure 1D**). SpiB protein expression was also decreased in the iGB-21 cells as compared to iGB-4 cells (**Figure 1E**). Co-staining of SpiB protein with memory/naive B cell marker (CD38) and PC marker (CD138) revealed that SpiB^high^ cells expressed higher levels of CD38 and little CD138, whereas SpiB^low^ cells expressed lower CD38 and were mostly CD138^+^ (**Figure 1E**). Among the iGB-4 cells, IgG1^+^ cells were largely SpiB^+^ CD38^high^ CD138^–^, whereas IgE^+^ cells contained a substantial population of SpiB^–^ CD38^low^ CD138^+^ cells, which markedly increased in iGB-21 cells, either of IgG1^+^ or IgE^+^ (**Supplementary Figures 2D, E**). This result corresponded to the notion that IgE^+^ B cells rapidly differentiate into PCs (26, 27) and IL-21 facilitates PC differentiation (7). These results suggest that SpiB may have a function to suppress PC differentiation while inducing the phenotype of B_mem_ cells.

### 3.2 SpiB suppresses PC differentiation while inducing the expression of Bach2 *in vitro*

**Figure 2:**
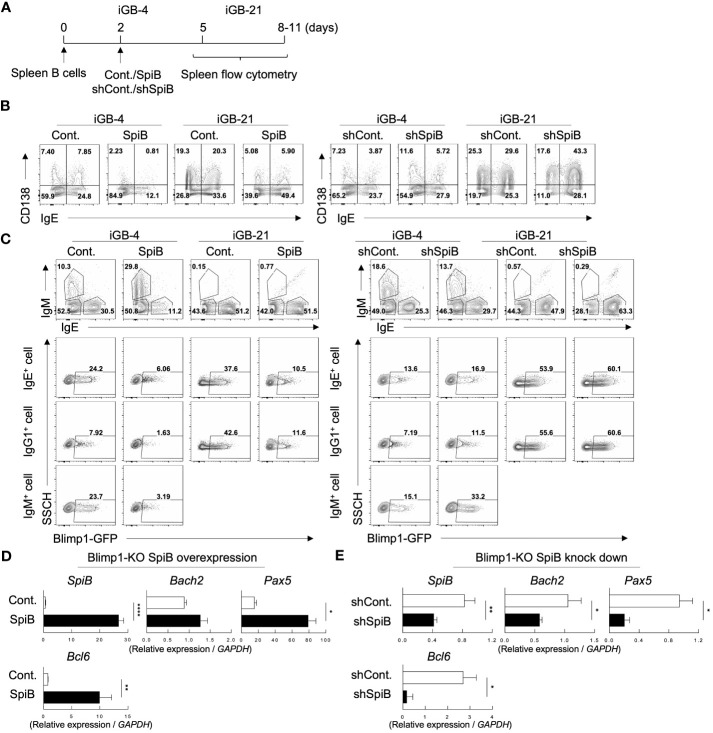
SpiB suppresses PC differentiation and *Blimp1* expression while upregulates *Bach2* expression *in vitro.* **(A)** Experimental protocol for retroviral overexpression and knockdown of SpiB in iGB cells. Spleen B cells were cultured on 40LB feeder cells with IL-4 for 5 days (IGB-4), to which the overexpression or knockdown retroviral vectors were introduced on day 2, then followed by the culture with IL-21 for 3- 5 days (iGB-21). **(B)** FCM analysis of CD138 and IgE expression on SpiB-overexpressing (left) or SpiB-knockdown (right) IGB-4 and iGB-21 cells. **(C)** FCM analysis of Blimp1-GFP expression in SpiB-overexpressing (left) or SpiB-knockdown (right) IGB-4 and iGB-21 cells. IgE^+^, IgM^+^ and IgE^-^ IgM^-^ (mostly IgG1^+^) populations were gated on iGB-4 and iGB-21 cells (top panels) and the expression of GFP in each population (bottom) were analyzed by FCM (bottom). **(D, E)** qRT-PCR analysis of mRNA expression of B cell-related genes in SpiB-overexpressng **(D)** and SpiB-knockdown **(E)** Blimp1-KO iGB-4 cells. *p < 0.05, **p < 0.01, ****p < 0.0001.

To better understand the function of SpiB in B cell differentiation, we performed retroviral overexpression or knockdown of SpiB in iGB cells (**Figure 2A**). Overexpression of SpiB resulted in the decrease, while knockdown of SpiB resulted in the increase, of the proportion of CD138^+^ cells in both IgE^–^ (mostly IgG1^+^) and IgE^+^ fractions of iGB-4 and iGB-21 cells (**Figure 2B**; **Supplementary Figure 3A**). PC differentiation is known to be induced by a transcription factor Blimp1 which is coded by the gene *Prdm1* (28). To further elucidate the mechanism of SpiB function, we analyzed the expression of *Prdm1* gene by using iGB cells derived from Blimp1-reporter (Blimp1-GFP) mice. Overexpression of SpiB resulted in the decrease, whereas knockdown of SpiB resulted in the increase, of the proportion of Blimp1 GFP^+^ cells within IgE^+^, IgM^+^ and IgE^–^ IgM^–^ (mostly IgG1^+^) B cells in iGB-4 and iGB-21 cells (**Figure 2C**). These results suggest the function of SpiB to suppress Blimp1 expression at a transcription level and thus PC differentiation.

We further analyzed the mRNA expression levels of B-cell-related genes in the iGB-4 cells over-expressing SpiB and confirmed that the expression of *Prdm1* was suppressed by SpiB (**Supplementary Figure 3B**). Of note, SpiB overexpression resulted in upregulation of the expression of *Bach2*, *Pax5*, and *Bcl6*, which encode transcription factors known to reciprocally suppress the expression of Blimp1 (6, 29–31) (**Supplementary Figure 3B**). Blimp1 is known to suppress several B cell-related genes to positively regulate PC differentiation (32–34). To clarify whether the SpiB-mediated upregulation of *Bach2*, *Pax5*, and *Bcl6* is independent of Blimp1, we used B cells from B-cell-specific Blimp1-deficient mice (Blimp1^f/f^ CD19-cre; here called Blimp1-KO mice) for iGB cell culture. Overexpression of SpiB in the Blimp1-KO iGB-4 cells resulted in an increase of the expression of *Bach2*, *Pax5*, and *Bcl6*, whereas knockdown of SpiB resulted in the opposite outcome (**Figures 2D, E**). These results suggest that SpiB functions to suppress the expression of *Blimp1* and independently induce *Bach2*, *Pax5*, and *Bcl6* in B cells.

### 3.3 SpiB facilitates B_mem_ cell maintenance in mice

**Figure 3:**
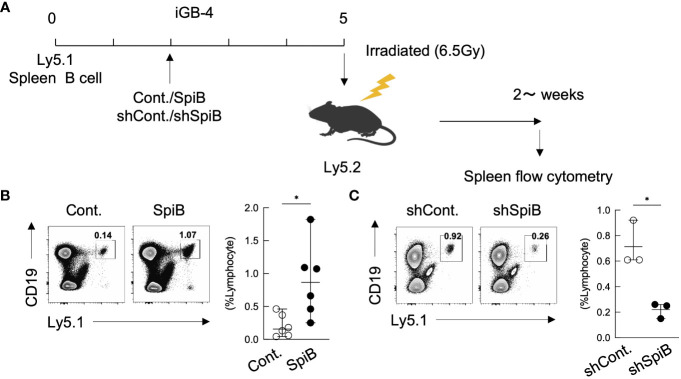
SpiB is critical for B_mem_ cell generation from iGB cells *in vivo.* **(A)** Experimental protocol for the generation of iMB cells from gene-transduced iGB-4 cells. iGB-4 cells derived from Ly5.1 congenic C57BL/6 (B6) mice were transduced with retroviral vectors expressing SpiB or shSpiB, or empty vectors as a control (cont.). These iGB-4 cells on day 5 were adaptively transferred to irradiated Ly5.2 congenic B6 mice. Spleen cells were analyzed 4 weeks later (for SpiB overexpression) or 2 weeks later (for SpiB knockdown) by FCM. **(B, C)** Frequencies of CD19^+^ Ly5.1^+^ iMB cells in the spleens of the recipient mice. A representative FCM data (left) and the frequencies among lymphocytes (right) [**(B)**: n = 6; **(C)**: n = 3]. *p < 0.05.

The above results indicating that SpiB suppresses the expression of Blimp1 while inducing the expression of Bach2, suggesting that SpiB functions to generate or maintain B_mem_ cells. To address this possibility, we applied our system in which iGB-4 cells differentiate to memory-like B (iMB) cells in the recipient mice (7). We retrovirally overexpressed or knocked down SpiB in iGB-4 cells and transferred these cells to irradiated mice respectively to generate iMB cells (**Figure 3A**). SpiB overexpression resulted in an increase in the frequency of the iMB cells (**Figure 3B**). On the contrary, SpiB knockdown resulted in the reduced frequency of the iMB cells (**Figure 3C**). These data indicated that SpiB promoted the generation or maintenance of iMB cells in a dose-dependent manner. These results strongly suggest that SpiB is a factor that regulates the generation or maintenance of B_mem_ cells.

**Figure 4:**
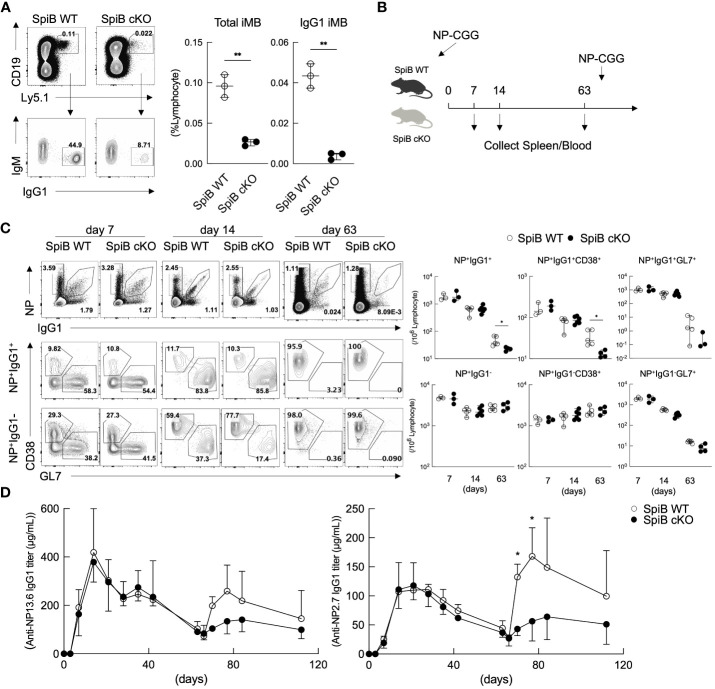
Long-lived B_mem_ cells and memory antibody response are defective in SpiB cKO mice. **(A)** Frequencies of CD19^+^ Ly5.1^+^ (total iMB) cells and CD19^+^ Ly5.1^+^ IgG1^+^ iMB cells in the spleens of the recipient mice transferred with iGB-4 cells derived from SpiB WT or SpiB cKO mouse B cells 4 weeks earlier, analyzed by FCM. A representative FCM data (left) and the frequencies among lymphocytes (right) (N =3). **(B)** A protocol of a mice immunization experiment. SpiB WT and SpiB cKO mice were intraperitoneally immunized with NP-CGG in alum and boosted with NP-GCC without alum to induce memory response at 66 days after the primary immunization. Spleen cells and blood were collected before immunization and at the indicated timepoints (spleen) and as indicated in **(D)** (blood). **(C)** Frequencies of NP^+^ IgG1^+^ or NP^+^ IgG1^-^, total B, B_mem_ and GC B cells in the spleens at the indicated time points after NP-CGG immunization in SpiB WT or SpiB cKO mice. Spleen cells were gated on B220^+^ Igκ^low^, NP^+^ IgG1^+^ or NP^+^ IgG1^-^ cells before gating on B_mem_ (GL7^-^ CD38^+^) or GC B (GL7^+^ CD38^-^) cells. A representative FCM data (left) and the frequencies among lymphocytes of three to six mice (right) are shown. **(D)** Concentrations of serum antibodies of total anti-NP-IgG1 (left) and high-affinity anti-NP-IgG1 (right) from SpiB WT or SpiB cKO mice primarily immunized with NP-CGG in alum and secondarily with NP-CGG alone on day 66. (n = 4) *p < 0.05, **p < 0.01.

To confirm this, we next analyzed the generation of B_mem_ cells from SpiB-deficient B cells. Since SpiB null-knockout mice were previously reported to have defective GCs (15), we analyzed SpiB-floxed (*SpiB* ^f/f^) mice (see Materials and Methods 2.2) crossed with Cγ1-cre mice expressing cre recombinase only in B cells class-switched to IgG1 (20) (here called ‘SpiB cKO’ mice). *SpiB* ^+/+^ Cγ1-cre (called ‘SpiB WT’) mice were used as controls. The deletion of SpiB was confirmed in IgG1^+^ B cells in the immunized mice and in iGB cells (**Supplementary Figures 4A–E**). We first examined the iMB cell generation from SpiB cKO iGB-4 cells *in vivo*. As expected from the result of SpiB-knockdown experiments (**Figure 3C**), very few IgG1^+^ iMB cells were generated from SpiB cKO iGB-4 cells (**Figure 4A**).

Next, we analyzed T cell-dependent (TD) immune response in the SpiB cKO mice by immunizing the mice with a typical TD antigen, NP-CGG, precipitated in alum. On day 7, 14, and 63 after the primary immunization, we analyzed GC B cells (GL7^+^ CD38^–^) and B_mem_ cells (GL7^–^ CD38^+^) within NP-specific IgG1^+^ and IgG1^–^ B cells by FCM (**Figure 4B**). The frequency and the absolute number of GC B and B_mem_ cells did not significantly differ between SpiB WT and SpiB cKO mice in both NP^+^ IgG1^+^ and NP^+^ IgG1^–^ B cell populations at 7- and 14-days post-immunization (**Figure 4C**). The concentration of blood anti-NP-IgG1 antibody was also similar between the two strains during the primary immune response (**Figure 4D**). On day 63 post-immunization, when GC B cells almost disappeared, the number of B_mem_ cells in the NP^+^ IgG1^+^ population, but not in the NP^+^ IgG1^–^ population, was significantly reduced in SpiB cKO mice as compared to SpiB WT mice. We further analyzed the memory antibody response in these mice to determine the effect of the reduction of IgG1^+^ B_mem_ cells. After re-immunization of the mice with NP-CGG without adjuvants, the generation of serum anti-NP IgG1 antibodies was attenuated in SpiB cKO mice during the secondary immune response, which was highlighted when we detected only high-affinity antibodies that are mostly produced from B_mem_ cells (**Figure 4D**). These results suggest that SpiB functions to maintain, rather than to induce, the antigen-specific B_mem_ cells.

### 3.4 Regulation of B-cell-related genes by SpiB in GC and B_mem_ cells

**Figure 5:**
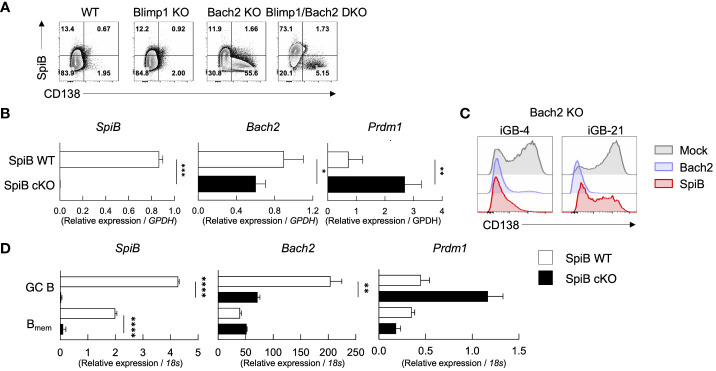
SpiB suppresses PC differentiation independently of Bach2. **(A)** Expression of intracellular SpiB and cell-surface CD138 in SpiB WT, Blimp1-KO, Bach2-KO and Blimp1/Bach2 DKO iGB-4 cells analyzed by FCM. **(B)** mRNA expression of *SpiB*, *Bach2*, and *Prdm1* in SpiB WT and SpiB cKO iGB-4 cells analyzed by qRT-PCR. **(C)** FCM analysis of CD138 expression on Bach2-KO iGB-4 (left) and iGB-21 (right) cells transduced with mock-, Bach2-, SpiB-expressing retroviral vectors. **(D)** qRT-PCR analysis for mRNA expression of *SpiB*, *Bach2*, and *Prdm1* in IgG1^+^ GC B and B_mem_ cells from spleens of SpiB WT and SpiB cKO mice immunized with NP-CGG in alum 14 days earlier. *p < 0.05, **p < 0.01, ***p < 0.001, ****p < 0.0001.

It is known that Bach2 functions as a suppressor for Blimp1 and regulates B_mem_ cell differentiation (6, 35). Our *in-vitro* analysis demonstrated that SpiB functions to suppress PC differentiation by suppressing Blimp1 expression while inducing the expression of Bach2 (**Figures 2C–E**). To elucidate the mechanism of SpiB function in B cells, we focused on the relationship of SpiB and Bach2. We first analyzed expression of SpiB and CD138 in the iGB-4 cells derived from splenic B cells of normal B6 (WT), Blimp1-KO, Bach2-KO, and Blimp1/Bach2-double KO mice by FCM. As expected, CD138^+^ cells were induced in Bach2 KO iGB cells. However, the induction of CD138^+^ cells was strongly suppressed in Blimp1/Bach2 double KO iGB-4 cells irrespective of their BCR isotype, indicating that Bach2 suppresses PC differentiation through Blimp1 suppression (**Figure 5A**; **Supplementary Figure 5A**).

We next analyzed the expression of *Bach2* and *Prdm1* along with other B cell-related genes, such as *Bcl6*, *Pax5* and *IRF4*, in SpiB cKO and SpiB WT iGB-4 cells. SpiB-deficiency resulted in decrease of the expression of *Bach2* while increase of *Prdm1* and *IRF4* (**Figure 5B**, **Supplementary Figure 5B**). To verify the functional meaning of the SpiB-regulated expression of Bach2 and Blimp1, we retrovirally transduced SpiB and Bach2 into Bach2 KO iGB cells. The frequency of CD138^+^ cells was strongly suppressed by SpiB overexpression in Bach-2 KO iGB-4 cells as observed by Bach2 reconstitution, and to a lesser extent in iGB-21 cells, indicating that SpiB can suppress PC differentiation independently of Bach2 (**Figure 5C**). To further analyze the relation between Bach2 and SpiB *in vivo*, we sorted IgG1^+^ GC and early B_mem_ cells from SpiB WT and SpiB cKO mice 14 days after immunization with NP-CGG in alum, and analyzed the expression of *Bach2*, *Prdm1* and other B cell-related genes. In GC B cells, the expression of *Bach2* was decreased while that of *Prdm1* was increased by the SpiB-deficiency, with other B cell-related genes largely unaffected, resembling the result with iGB-4 cells. In the early B_mem_ cells, however, the expression of *Bach2* and *Prdm1*, as well as of other genes, was not apparently affected by the SpiB-deficiency (**Figure 5D**; **Supplementary Figure 5C**). These results indicate that SpiB upregulates *Bach2* expression and suppresses *Blimp1* expression and PC differentiation independently of Bach2 in GC B cells, which may render the longevity to the descendant B_mem_ cells.

### 3.5 SpiB upregulates anti-apoptosis and autophagy genes and supports survival in B_mem_ cells

**Figure 6:**
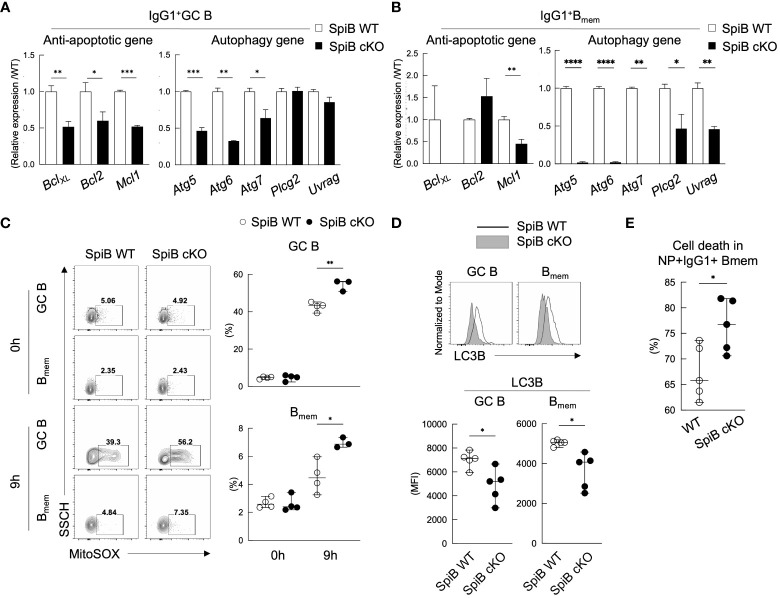
SpiB-deficient GC B and B_mem_ cells express lower levels of anti-apoptotic and autophagy genes and are more prone to die. **(A, B)** qRT-PCR analysis for mRNA expression of indicated anti-apoptotic genes (left) and autophagy genes (right) in IgG1^+^ GC B cells **(A)** and IgG1^+^ B_mem_ cells **(B)** from spleens of SpiB WT and SpiB cKO mice immunized with NP-CGG in alum 14 days earlier. **(C)** FCM analysis for MitoSOX staining of the same cells as in **(A, B)** Cells were incubated for 0 or 9 hours in non-FCS medium before staining. Representative FCM data (left) and the frequencies of MitoSOX-positive cells (n = 3 or 4) (right). **(D)** FCM analysis for the expression of LC3B in the same cells as in **(A, B)** Representative FCM data (top) and mean fluorescence intensity (MFI) of the LC3B stained cells (bottom) are shown (n = 5). **(E)** Frequencies of dead cells in NP^+^ IgG1^+^ CD38^+^ B_mem_ cells from spleens of SpiB WT and SpiB cKO mice immunized with NP-CGG in alum 14 days earlier. Percentages of the dead cells detected microscopically following Trypan Blue staining after 9 hours’ culture in non-FCS medium as compared to those before the culture (n = 5). *p < 0.05, **p < 0.01, ***p < 0.001, ****p < 0.0001.

Since the phenotype of SpiB cKO mice in the TD immune response was apparent only in the late B_mem_ cells and their recall response, we suspected the function of SpiB to be more related to the maintenance of B_mem_ cells rather than their generation. Previous studies have shown that autophagy is one of the mechanisms to inhibit apoptosis and to support the survival of memory T and B cells (36–39). Therefore, we analyzed the expression levels of anti-apoptotic genes and autophagy genes in GC B and B_mem_ cells sorted from SpiB cKO and SpiB WT mice 14 days after immunization with NP-CGG. As a result, SpiB cKO GC B cells express lower levels of anti-apoptotic genes (*Bcl-X_L_*, *Bcl2*, *Mcl1*) and some autophagy genes (*Atg5*, *Atg6*, *Atg7*) as compared to SpiB WT GC B cells and the difference was more profound in B_mem_ cells, except for *Bcl2* (**Figures 6A, B**).

Autophagy is known to be necessary for the clearance of dysfunctional mitochondria that produce mitochondrial reactive oxygen species (mROS) (40) which is one of the factors to induce apoptosis (41). Given the almost undetectable levels of the autophagy genes, *Atg5*, *Atg6*, *Atg7*, in SpiB cKO B_mem_ cells, we assessed mROS production in GC B and B_mem_ cells sorted from immunized SpiB WT and SpiB cKO mice by staining them with MitoSox and analyzing by FCM. MitosSox positive cell population was small and similar between SpiB WT and SpiB cKO GC B and B_mem_ cells before culture (0 h), but the frequency of MitoSox-positive cells increased after 9 hours’ culture more abundantly in SpiB cKO GC B and B_mem_ cells than SpiB WT ones (**Figure 6C**). We next addressed autophagy activity in SpiB cKO GC B and B_mem_ cells by evaluating the expression of a known autophagy marker LC3B (42–44). When compared with those from SpiB WT mice, GC B and B_mem_ cells from SpiB cKO mice showed reduced expression of LC3B, indicating lower activity of autophagy in the absence of SpiB in these cells (**Figure 6D**). Finally, we found that IgG1^+^ B_mem_ cells in the immunized SpiB cKO mice contained more dead cells than those in the immunized SpiB WT mice after 9-hour incubation in the plain medium (**Figure 6E**). Taken together, these results indicate that the lack of SpiB in GC B and B_mem_ cells results in reduction of gene expression and activity for autophagy, leading to the accumulation of mROS and increased susceptibility to apoptosis.

## 4 Discussion

Having been faced with the global pandemic of SARS-CoV-2, fundamental regulation of B_mem_ cell survival after vaccination is gathering more attention than ever. How B_mem_ cells behave after the re-exposure to the antigen has recently becoming clearer. For example, B_mem_ cell subsets characterized by BCR isotypes as well as the expression of CD80/PD-L2 were shown to behave differently in the recall response in both human and mice (45, 46). However, mechanisms of B_mem_ cell generation and maintenance are largely unknown. While it was reported that Bach2 is required for the generation of B_mem_ cells, Bach2 was expressed in majority of GC B cells as well as naive B cells (6). Thus, the specific function of Bach2 in the B_mem_ cell development remains obscure.

To tackle this difficult problem, we used our original *in-vitro*-induced GC B (iGB) cell culture and induced memory B (iMB) cell system for the functional analysis of transcription factors by retroviral overexpression and knockdown. In this study, we focused on transcription factors whose expression levels were higher in the B_mem_-like iMB cells than naive B cells, and identified SpiB that acts as a key factor for the maintenance of B_mem_ cells. Overexpression of SpiB suppressed differentiation of CD138^+^ PCs by inhibiting the expression of Blimp1, while knockdown of SpiB facilitated the PC differentiation, in the iGB cells *in vitro*. We further assessed *in-vivo* development of iMB cells from these manipulated iGB cells. Overexpression of SpiB in iGB cells increased the frequency of iMB cells, while knockdown of SpiB decreased it. These results indicated the role of SpiB in B_mem_ cell generation and/or maintenance well before generation and analysis of SpiB KO mice, demonstrating the iGB-iMB system as a powerful and easier tool to investigate the molecular mechanisms for GC B cell differentiation *in vivo*. In accord with this result, the number of long-lived B_mem_ cells was significantly reduced in SpiB cKO mice, consistent with the supposed role of SpiB in B_mem_ cell maintenance.

The mechanism of the maintenance of B_mem_ still remains unclear. It has been supposed that anti-apoptotic protein Bcl2 promotes survival of B_mem_ cells mainly from the results showing increased number of B_mem_ cells in Bcl2-transgenic mice. However, it has been reported that transgenic Bcl2 inhibits apoptosis and promotes proliferation of GC B cells, resulting in an increased number of B_mem_ cells (47, 48). Therefore, the contribution of Bcl2 to B_mem_ cell survival will remain unclear until the B_mem_-cell-specific Bcl2-knockout mice become available. Our data demonstrated that the expression of Bcl2 gene was not reduced in the SpiB-deficient B_mem_ cells being affected in their long-liveness, instead the expression of BclxL gene was almost abrogated. Although BclxL was reported to be dispensable for development of GC B and B_mem_ cells (49), BclxL may play a redundant role in collaboration with other factors that are regulated by SpiB.

It has been reported that autophagy protein Atg7 regulates B_mem_ cell survival (39, 50, 51) and that the Atg7-dependent autophagy is independent of Bcl2-mediated anti-apoptosis pathway (39, 51). We found that the expression of autophagy genes, *Atg5, Atg6*, and *Atg7* was markedly reduced in the SpiB-deficient B_mem_ cells, which exhibited a phenotype with diminished autophagy activity, increased mitochondrial ROS, and augmented cell death. Thus, attenuated mitophagy resulting in the over-production of ROS, together with the abolished BclxL expression, may render B_mem_ cells prone to die earlier in SpiB-KO mice.

Direct binding and regulation by SpiB of its possible target genes in GC B cells remain to be determined, for example, by ChIP-Seq analysis. Regarding this, it was reported that PU.1 and SpiB share some binding targets in B cell lymphoma, suggesting that they may work together as co-factors to regulate target genes in GC B cells (52, 53). In mice lacking both PU.1 and SpiB in B cells, the number of follicular B cells was decreased and the GC response was abrogated, while these defects were ameliorated in mice lacking PU.1 alone in B cells, and not apparent in mice lacking SpiB alone (53). This result suggests that PU.1 is mainly responsible for the generation of follicular and GC B cells, but SpiB compensates this function to some extent when PU.1 is absent. Thus, SpiB does not seem to be responsible for these functions but for the maintenance of B_mem_ cells under normal conditions.

In conclusion, we found that SpiB plays a critical role in the maintenance of B_mem_ cells. Further studies are needed to fully understand the mechanisms underlying SpiB-mediated B_mem_ cell maintenance and the potential therapeutic implications of this finding. Investigating of the detailed function of SpiB and its relationship with genes involved in metabolism, will provide a better understanding of the regulation of B_mem_ cells, ultimately leading to the development of vaccines that can generate longer-lasting humoral memory.

## Data availability statement

The raw data supporting the conclusions of this article will be made available by the authors, without undue reservation.

## Ethics statement

The animal study was approved by Animal Care and Use Committee of Tokyo University of Science. The study was conducted in accordance with the local legislation and institutional requirements.

## Author contributions

DK supervised and funded the project. SH designed and performed the experiments. TKo and HT contributed to conducting the experiments. KH, IS, YF-O and TKa were involved in methodology, investigation, and writing of the original draft, on SpiB cKO mice. SH and DK wrote the manuscript. All authors contributed to the article and approved the submitted version.
