# Supplementary material for: SpiB regulates the expression of B-cell-related genes and increases the longevity of memory B cells
Source: Front Immunol. 2023 Oct 27;14:1250719. doi: 10.3389/fimmu.2023.1250719 (PMC10641807; doi:10.3389/fimmu.2023.1250719)
Supplement: Supplementary file 1 [file DataSheet_1.pdf]

Supplementary Material

SpiB regulates the expression of B-cell-related genes and increases the longevity of memory B cells

Shu Horiuchi[1†], Takuya Koike[1†], Hirofumi Takebuchi[1], Katsuaki Hoshino[2,3], Izumi Sasaki[4], Yuri Fukuda-Ohta[4], Tsuneyasu Kaisho[3,4], Daisuke Kitamura[1*]

[1]Division of Cancer Cell Biology, Research Institute for Biomedical Sciences, Tokyo University of Science, Noda, Chiba, Japan

[2]Department of Immunology, Faculty of Medicine, Kagawa University, Miki-cho, Kagawa, Japan

[3]Laboratory for Human Disease Models, RIKEN Center for Integrative Medical Sciences, Yokohama, Kanagawa, Japan

[4]Department of Immunology, Institute of Advanced Medicine, Wakayama Medical University, Wakayama, Japan

*** Correspondence:**
Daisuke Kitamura
kitamura@rs.tus.ac.jp

[†]Present address:

Shu Horiuchi, CSL, Waltham, MA, USA;

Takuya Koike, Center for Infectious Disease Education and Research, Osaka University, Osaka, Japan.

Supplementary Figures 1 ~ 5

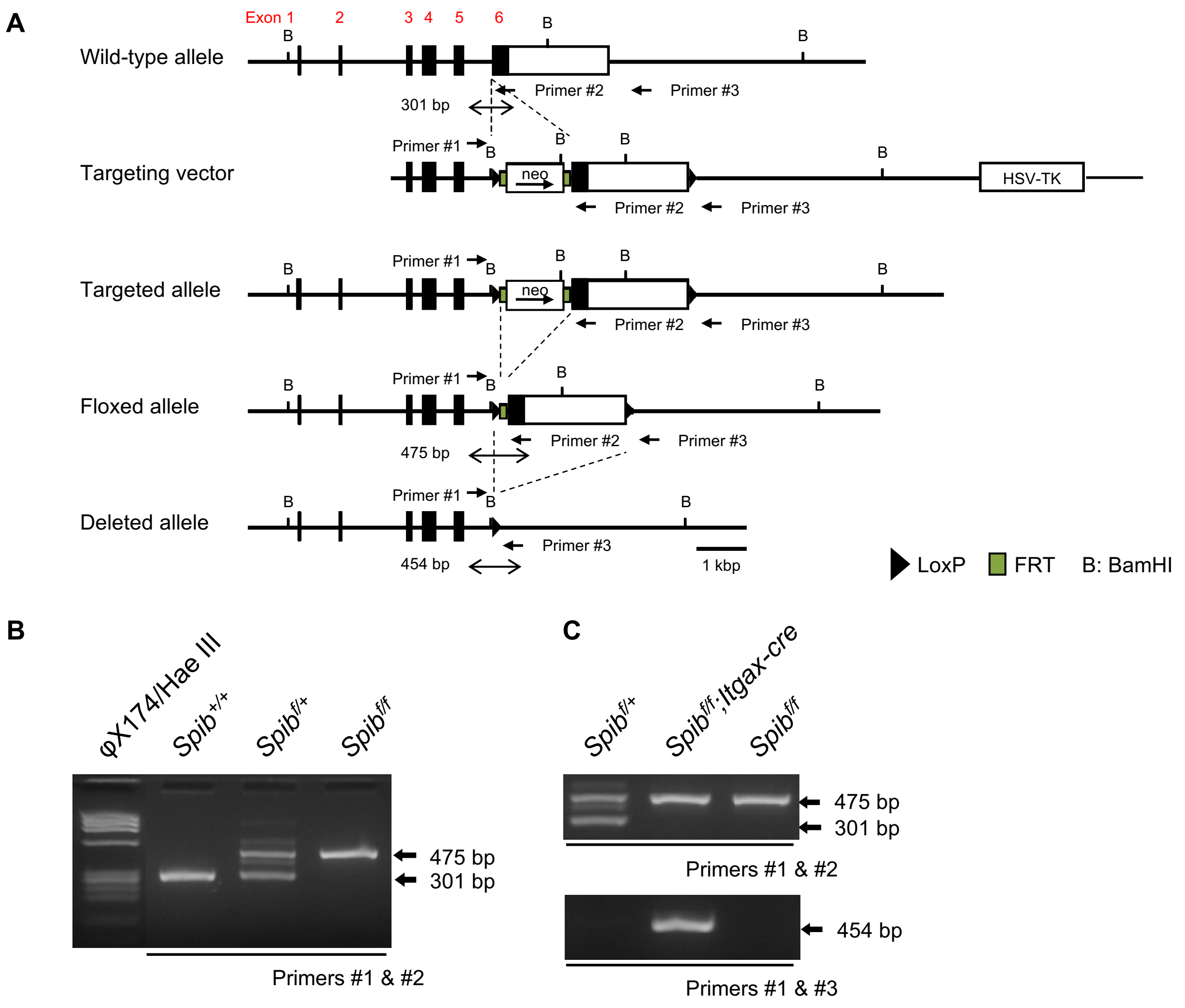

Supplementary Figure 1. Generation of *Spib* mutant alleles

(A) Scheme of the targeting vector and wild-type, targeted, floxed and deleted alleles of *Spib*. Closed boxes, coding exons; open box, non-coding exon. **(B, C)** PCR analysis of genomic DNA extracted from the tail samples. *Spib*$^{f/f}$; *Itgax-cre* mice are homozygous *Spib* floxed mice carrying the gene encoding the Cre recombinase driven by the *Itgax* promoter. The CD11c-Cre mouse (B6.Cg-Tg(Itgax-cre)1-1Reiz/J) was obtained from Dr. Boris Reizis (1).

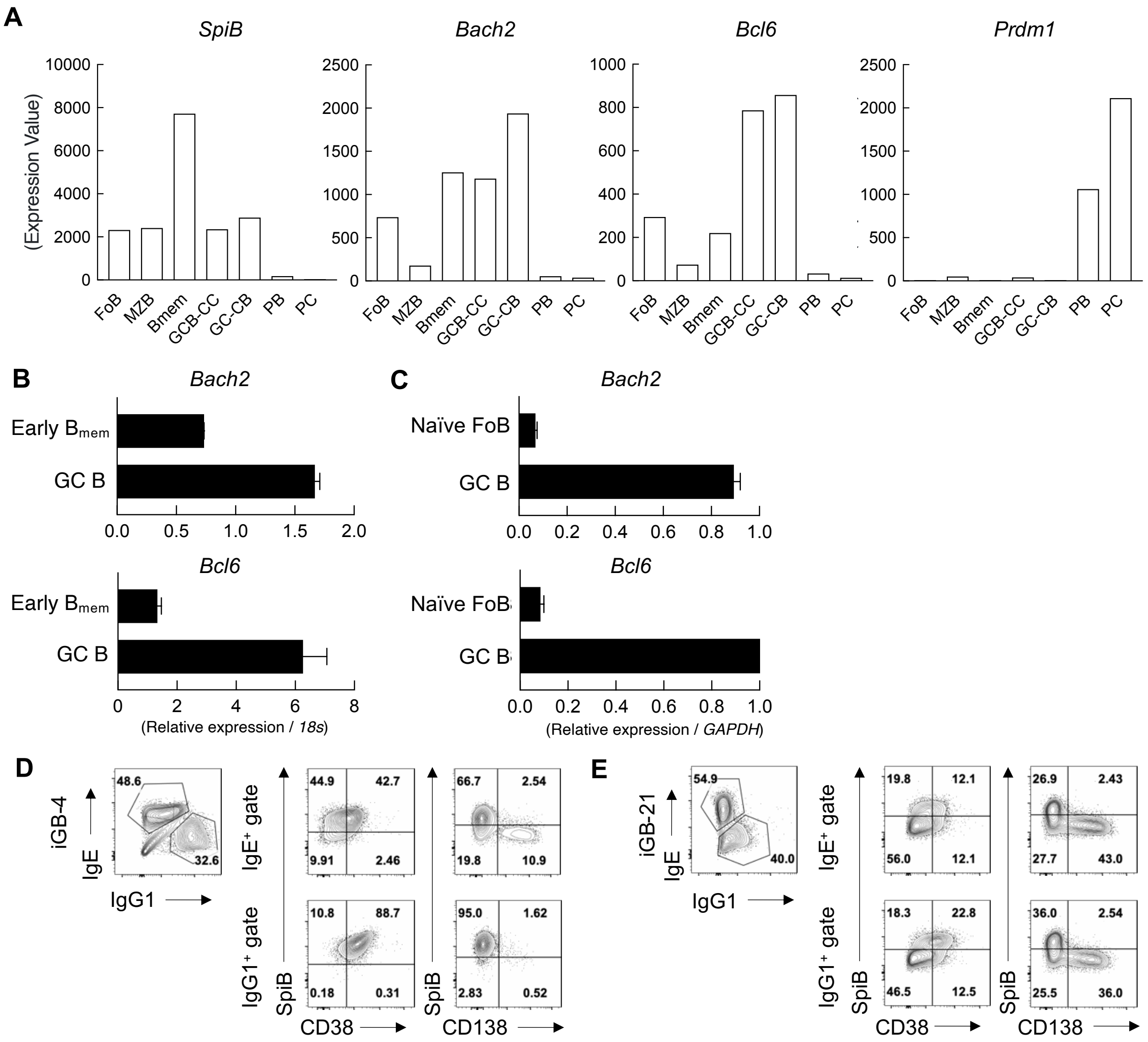

Supplementary Figure 2. SpiB is expressed in GC B and B_{mem} cells and induced in iGB cells

(A) mRNA expression of B-cell-related genes in follicular B cells (FoB), marginal zone B cells (MZB), B_{mem} cells, GC centrocytes (GCB-CC), GC centroblasts (GC-CB), plasmablasts (PB), plasma cells (PC) from mouse spleens (summarized data from ImmGen). **(B, C)** mRNA expression of *Bach2* (B) and *Bcl6* (C) in early B_{mem}, GC B and naive FoB cells sorted from spleens of immunized mice as described in Figure 1A. **(D, E)** FCM analysis for SpiB expression in iGB-4 (D) and iGB-21 (E) cells. The expression of IgG1 and IgE is shown in left. Expression of intracellular SpiB and cell-surface CD38 (middle) or CD138 (right) in each IgE^+ and $IgG1^+$ gated cells are shown.

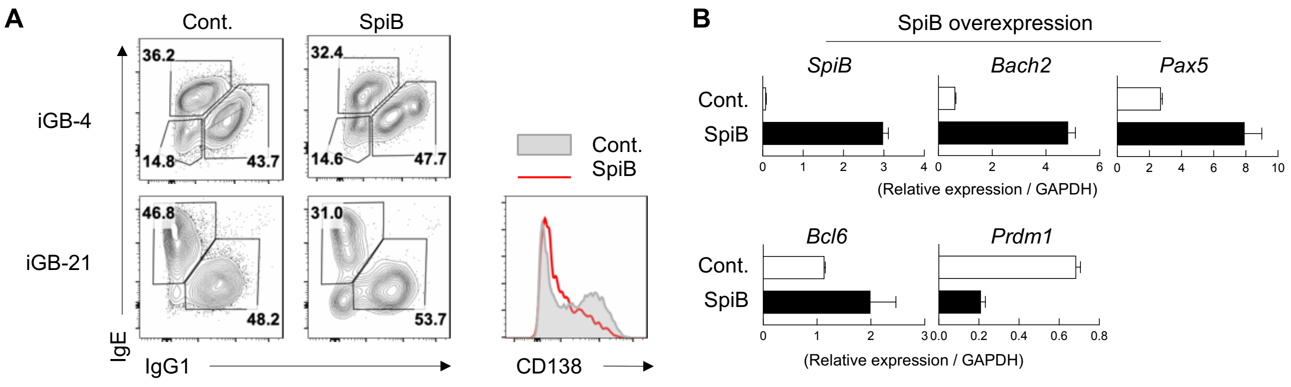

Supplementary Figure 3. SpiB overexpression suppresses the expression of *Prdm1* and plasma cells differentiation

(A) FCM analysis for the expression of IgG1 and IgE in iGB-4 (top) and iGB-21 (bottom) cells transduced with control (Cont., left) or SpiB-expressing (SpiB, middle) retrovirus vectors. The expression of CD138 on the control or SpiB-transduced iGB-21 cells are shown by histograms (right).

(B) qRT-PCR analysis for the mRNA expression of B cell-related genes in the control or SpiB-transduced iGB-4 cells.

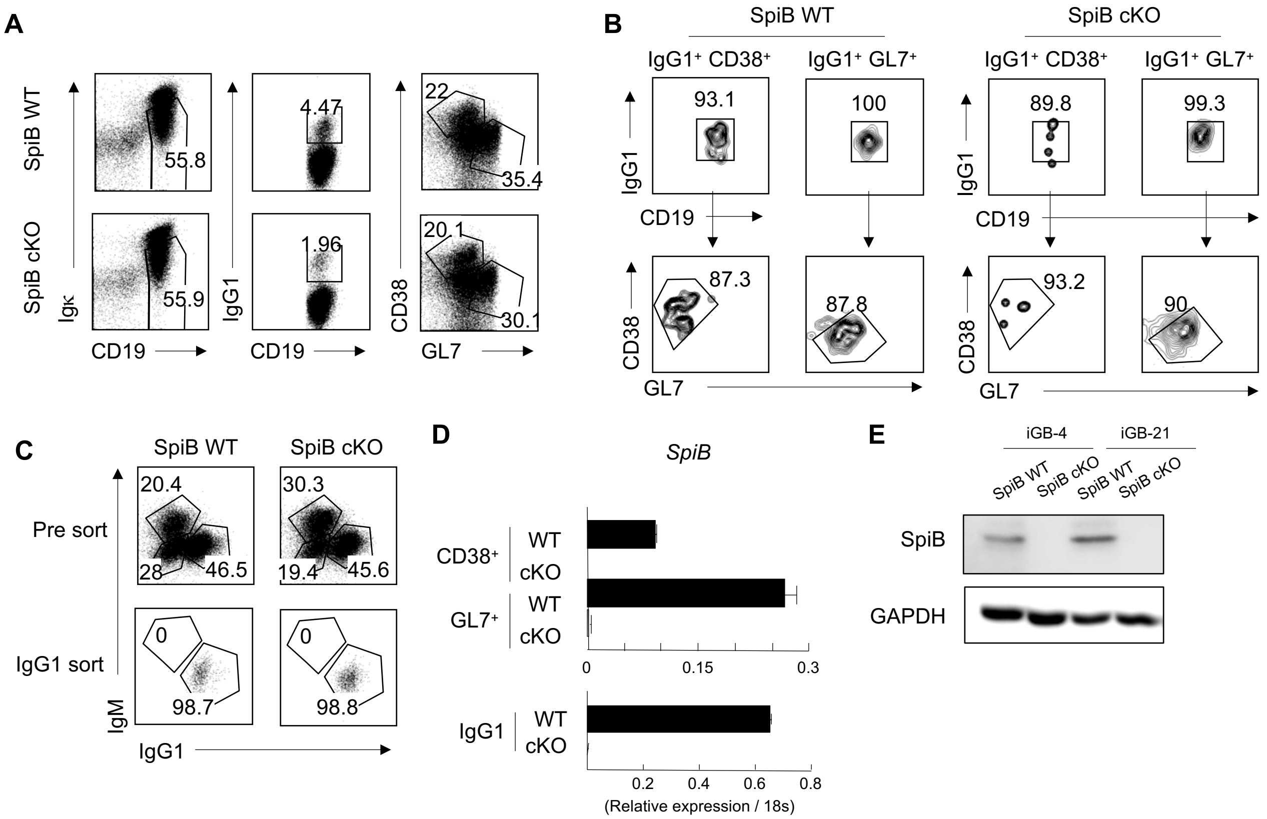

Supplementary Figure 4. Evaluation of SpiB expression in IgG1 B cells of SpiB cKO mice

(A) Sorting strategy of IgG1$^+$ GC B and B$_{mem}$ cells from spleens of SpiB cKO and SpiB WT mice immunized with NP-SRBC 7 days earlier. The gating strategy for GC B cells (CD19$^+$ Igκ$^-$ IgG1$^+$ GL7$^+$ CD38$^-$) and early B$_{mem}$ cells (CD19$^+$ Igκ$^-$ IgG1$^+$ GL7$^-$ CD38$^+$) is shown. **(B)** FCM analysis of the cells sorted as in A. **(C)** Sorting of IgG1$^+$ cells from iGB-4 cells derived from WT or SpiB cKO mouse B cells. A representative result of pre-sort (top) and post-sort (bottom) cells are shown. **(D)** *SpiB* mRNA expression in IgG1$^+$ early B$_{mem}$ (CD38$^+$) and GC B (GL7$^+$) cells sorted from SpiB WT and SpiB cKO mice (top), or IgG1$^+$ iGB-4 (bottom), sorted as in A,B or C, respectively. **(E)** WB detection of SpiB and GAPDH (loading control) in the lysates of iGB-4 and iGB-21 cells derived from spleen B cells of WT and SpiB cKO mice.

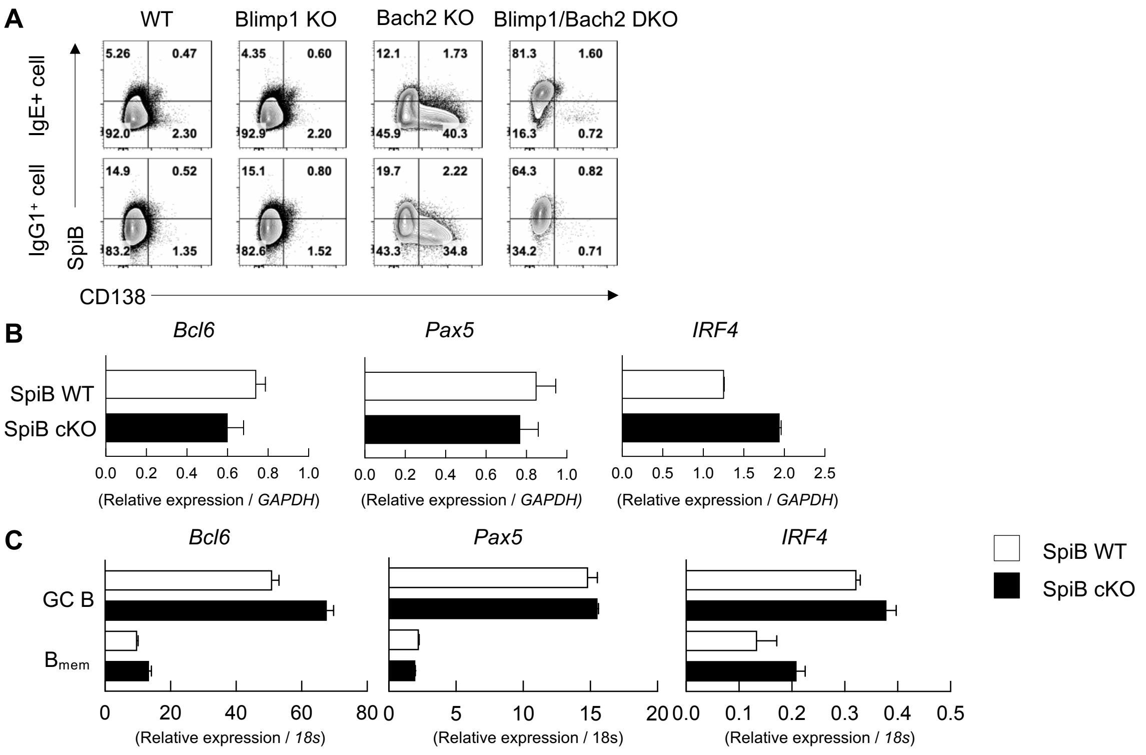

Supplementary Figure 5. SpiB suppresses plasma cell differentiation independently of Bach2
(A) FCM analysis for the expression of intracellular SpiB and cell-surface CD138 in iGB-4 cells gated on IgE^+ cells (top) or $IgG1^+$ cells (bottom). The iGB-4 cells were derived from spleen B cells of normal B6 (WT), Blimp1-KO, Bach2-KO or Blimp1/Bach2-DKO mice. **(B)** qRT-PCR analysis for the mRNA expression of the indicated genes in iGB-4 cells derived from spleen B cells of SpiB WT and SpiB cKO mice. **(C)** qRT-PCR analysis for the mRNA expression of the indicated genes in GC B and B_{mem} cells sorted from spleens of immunized SpiB WT and SpiB cKO mice, as in Figure 5D.

References

1. Caton ML, Smith-Raska MR, Reizis B. Notch-RBP-J signaling controls the homeostasis of CD8- dendritic cells in the spleen. J Exp Med. 2007;204(7):1653-1664.
